# New Zr-Ti-Nb Alloy for Medical Application: Development, Chemical and Mechanical Properties, and Biocompatibility

**DOI:** 10.3390/ma13061306

**Published:** 2020-03-13

**Authors:** Oleg Mishchenko, Oleksandr Ovchynnykov, Oleksii Kapustian, Maksym Pogorielov

**Affiliations:** 1 NanoPrime, 25 Metalowcow Str., Dedice 39-200, Poland; Dr.Mischenko@i.ua; 2 Department of Surgical and Propaedeutic Dentistry, Zaporizhzhia State Medical University, 26, Prosp.Mayakovskogo, Zaporizhzhia 69035, Ukraine; 3 Department of Physics and Engineering, Zaporizhzhia Polytechnic National University, 64 Zhukovsky Str, Zaporizhzhia 69063, Ukraine; aek@zntu.edu.ua (O.O.); aekzntu@gmail.com (O.K.); 4 Centre of Collective Use of Scientific Equipment, Sumy State University, 2 R-Korsakova Str, Sumy 40007, Ukraine

**Keywords:** Zr-Ti-Nb alloy, Young modulus, mechanical properties, biocompatibility

## Abstract

The concept of mechanical biocompatibilities is considered an important factor for orthopedics and dental implants. The high Young modulus of traditional Ti-based alloys can lead to stress-shielding syndrome and late postoperative complications. The development of new Al- and V-free Ti alloys with a low elastic modulus is a critical task for implantology. Despite the relatively low Young modulus and appropriate biological response of metastable beta-Ti alloys, their production requires complex metallurgical solutions and a high final cost that limit commercial application. The current research aimed to develop a Zr-Ti-Nb system with a low Young modulus suitable for biomedical application, including orthopedics and dental implantology. Two different charges were used for new alloy production with melting in a vacuum-arc furnace VDP-1 under atmospheric control (argon + helium) with a non-consumable tungsten electrode and a water-cooled copper crystallizer. Post-treatment included a forging-rolling process to produce a bar suitable for implant production. SEM with EDX and the mechanical parameters of the new alloy were evaluated, and a cell culture experiment provided a biocompatibility assessment. The chemical composition of the new alloy can be represented as 59.57-19.02-21.41 mass% of Zr-Ti-Nb. The mechanical properties are characterized by an extremely low Young modulus—27,27 GPa for the alloy and 34.85 GPa for the bar. The different master alloys used for Zr-Ti-Nb production did not affect the chemical compound and mechanical parameters so it was possible to use affordable raw materials to decrease the final price of the new product. The cell culture experiment demonstrated a full biocompatibility, indicating that this new alloy can be used for dental and orthopedics implant production.

## 1. Introduction

A high strength, low density, excellent corrosion resistance, high biocompatibility, and the ability of osseointegration are critical properties for metal alloys used for the production of dental and orthopedics implants [1]. Ti alloys have been used for this purpose for many years, with high clinical success [2], and due to their higher life expectancy, implants are now expected to serve for much longer periods. Additionally, the concept of mechanical biocompatibilities is considered an important factor [3,4]. Commercially pure titanium and the Ti–6Al–4V alloy are the most used metals for implant production, with a higher elastic stiffness and a relatively high elastic modulus (110 GPa) in comparison with cortical bone (from 15 to 30 GPa) [5]. However, toxic metal release (Al and V) from Ti–6Al–4V implants has been reported after long-term implantation [6].

A low modulus in dental implants can induce better stress transfer along the bone–implant interface and limit crestal bone loss due to excessive load transmitted to the bone through the crest module [7]. Some research has shown that decreasing the elastic modulus can minimize the bone atrophy due to the stress shielding effect, and increase the durability of orthopedic implants [8,9]. Based on these suggestions, the development of new Al- and V-free Ti alloys with a low elastic modulus is a critical task for implantology [10].

Metastable beta Ti-based alloys with nontoxic metals such as Nb and Zr displayed a reversible stress-induced martensitic transformation that led to a superelastic effect (high recoverable strain) and a very significant reduction of the apparent elastic modulus [11,12]. Some research has shown successful solutions for low-modulus alloy development. Liang S.X. and co-authors developed a β Ti alloy (Ti–31Nb–6Zr–5Mo, wt.%) using hot rolling followed by aging treatments, with a final Young modulus of around 48 GPa [13]. The Ti–24Nb–4Zr–8Sn alloy with a Young modulus of 49 GPa developed by Nune K.S. showed an additional stimulation of osteoblast proliferation [14]. The Ti-24Nb-4Zr-7.9Sn alloy developed by the Institute of Metal Research Chinese Academy of Sciences (elastic modulus—42 GPa) showed a good biocompatibility in both cell culture and animal models [15]. Despite their relatively low Young modulus and appropriate biological response, all mentioned alloys require complex metallurgical solutions and have a high final cost that limit their clinical application.

The current research aimed to develop a Zr-Ti-Nb system with a low Young modulus suitable for biomedical material application, including orthopedics and dental practice. To achieve all requirements for medical implants, we expected the development of a Zr-Ti-Nb system with 59.57 mass% (Zr), 19.02 mass% (Ti), and 21.41 mass% (Nb). The predicted mechanical properties expected were a tensile strength of 800–1300 MPa and elastic modulus of 40–70 GPa. Dental implant production requires the development of cylindrical bars with the following characteristics: 6.0 mm in diameter and 1.5–3.0 m in length, with Ra 1.6–3.2.

## 2. Materials and Methods

### 2.1. Materials

For the development of a Zr-Ti-Nb alloy with the predicted properties, we used two different charges with the following composition:

Charge 1—zirconium iodide (Zr-I), Niobium (NbSh), and Titanium Sponge (TiG-120);

Charge 2—zirconium CTZ-110, Niobium (NbSh) and Titanium Sponge (TiG-90).

Niobium (NbSh) was purchased from PJSC (Titanium Institute, Zaporizhzhia, Ukraine) and manufactured by metallothermal technology. Titanium Sponge (TiG-120) was obtained from Zaporizhzhia Titanium & Magnesium Combine Ltd. (Zaporizhzhia, Ukraine); Zirconium iodide and CTZ were purchased from SSE (Zirconium, Zaporizhzhia, Ukraine). Zirconium iodide has an extremely high price and zirconium CTZ-110 was used in charge 2 to decrease the final price of the Zr-Ti-Nb alloy. Taking into account that zirconium CTZ-110 has a higher oxygen concentration, Titanium Sponge TG-90 was used in charge 2 to equalize the oxygen amount in the final alloy. The element composition of feedstock is listed in Table 1, Table 2 and Table 3. 

### 2.2. Melting Process

Melting was carried out in a vacuum-arc furnace VDP-1 with an atmospheric controller (argon + helium), with a non-consumable tungsten electrode and a water-cooled copper crystallizer. The VDP-1 furnace was invented and produced by PJSC (Titanium Institute, Zaporizhzhia, Ukraine).

### 2.3. SEM and EDX

The structure and composition, as well as the distribution quality, of the main alloying elements, were evaluated using a JSM-IT300LV scanning microscope (Jeol, Tokyo, Japan) equipped with X-Max 80 X-ray energy dispersive microanalysis (Oxford Intruments, Abingdon, UK). The investigation was performed at an accelerating voltage of 15 kV and an electron probe diameter of 4 nm, while the diameter of the x-ray excitation zone was about 2 μm. The chemical composition was determined by the non-standard method of calculating fundamental parameters: by calculating the correction reflection coefficients of the electrons of the probe, the absorption of characteristic x-ray radiation, and fluorescence. Three test specimens were used for each measurement method.

### 2.4. X-ray Phase Analysis

The X-ray powder diffraction technique was used to characterize the phase composition and structural parameters. X-ray diffraction patterns were recorded using a standard powder diffractometer DRON-3 (Burevestnik, Moscow, Russia) with a Bragg-Brentano geometry and filtered Cu Kα radiation. Data were collected in the 2θ range from 20° to 120°, with a step interval of 0.05° and a count time of 5 sec per step.

### 2.5. Metallographic Investigations

Specimens were polished using a grinding wheel (grits 240–400–600 µm), with final polishing being conducted by a felt circle with a 3 µm diamond emulsion. Metallographic studies of the macro- and microstructure of the non-etched and etched state and analysis of fractographs of cast and deformed alloys were performed on the optical microscope "NEOPHOT-32" (Carl Zeiss Jena, Jena, Germany), as well as on the scanning electron microscope JSM-IT300LV (Jeol, Tokyo, Japan) at a magnification of X500—5000. For the analysis of the macrostructure, the grinders were etched in solution: 20% hydrofluoric and 20% nitric acids, with a glycerin base. Grinds for microstructure analysis were etched in a solution of hydrofluoric (20%) and nitric (20%) acids, with a glycerol base. Three test specimens were used for each measurement method.

### 2.6. Mechanical Parameters

The mechanical parameters of the developed alloy and final bar were investigated using the INSTRON 8801 machine (Instron, Norwood, MA, USA). To determine the mechanical properties, the following parameters were evaluated: tensile strength (σ_B_, MPa), yield strength (σ_O2_, MPa), elastic modulus (E, GPa), elongation (δ, %), and transverse narrowing (ψ, %). All of the experiments were conducted in triplicate.

### 2.7. Biocompatibility Study

The cell culture technique was used to assess the biocompatibility of the final Zr-Ti-Nb bar using a standard biocompatibility test [16]. Cylindrical specimens that were 6 mm in diameter and 5 mm high were prepared for the cell culture experiment. Samples were sterilized by 70% ethanol for 3 h at room temperature, washed in PBS twice, and then placed in 24-well plates. Dulbecco’s Modified Eagle Medium/Nutrient Mixture F-12 (DMEM/F-12) with L-glutamine, containing 100 units/mL penicillin, 100 µg/mL streptomycin, 2.5 µg/mL amphotericin B, 10% Fetal Bovine Serum, and 1.0 ng/mL bFGF, was added to each well. It was then incubated at 37 °C in a humidified environment with 5 % CO_2_. After 24 h, the human osteoblast cells (U2OS cells obtained from Umeo University, Umeo, Sweden) were seeded at 10^4^ cells per sample in 2 mL of DMEM/F-12. Test specimens with cells were incubated at 37 °C with 5% CO_2_, and media was changed every two days during a seven-day culture period. All of the experiments were conducted in triplicate.

The Alamar Blue (AB) assay was used to assess the cell viability on day one, three, and seven after seeding. The media was removed from each well and washed with PBS. One milliliter of Alamar Blue™ solution was added to each well and then incubated for two hours. Two aliquots of 200 µL of Alamar Blue™ solution were collected from each scaffold and the absorbance was read on the absorbance reader using 570 and 600 nm wave lengths.

### 2.8. Statistics

One-way ANOVA with multiple comparisons was used to assess the difference between groups using GraphPad Prism 8.0 software (V 8.0, San Diego, CA, USA). Statistical significance was assumed at a confidence level of 95% (*p* < 0.05).

## 3. Results and Discussion

### 3.1. Melting and Bar Preparation

**Figure 1:**
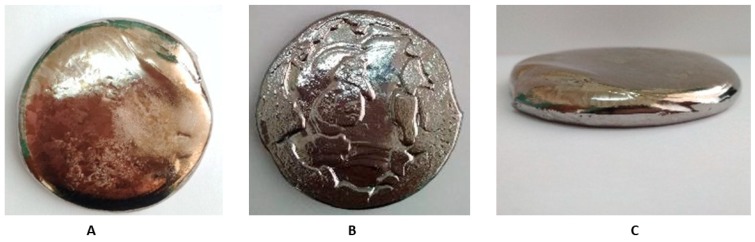
Zr-Ti-Nb ingot after arc melting—upper (**A**), lower (**B**), and lateral (**C**) view.

Master alloys were tabbed to the furnace simultaneously with tight packing. The mass of the charge was calculated for melting the alloy at the amount of 1.6 kg, which is necessary to obtain cylindrical billets with a diameter of 6 mm and a length of up to 3000 mm (taking into account processing losses). For a uniform distribution of alloying elements and impurities throughout the ingot volume, triple remelting of each ingot was performed. The melting process was performed with the following parameters: I = 400–600 A; U = 62–64 V; pre-vacuum = 1 × 10^−4^ mbar; and melting medium = argon (99.999 Ar) at pressure P = −0.5 kgf/cm^2^. After triple re-melting, we obtained the ingot used for bar preparation and the investigation of the chemical and mechanical parameters (Figure 1).

**Figure 2:**
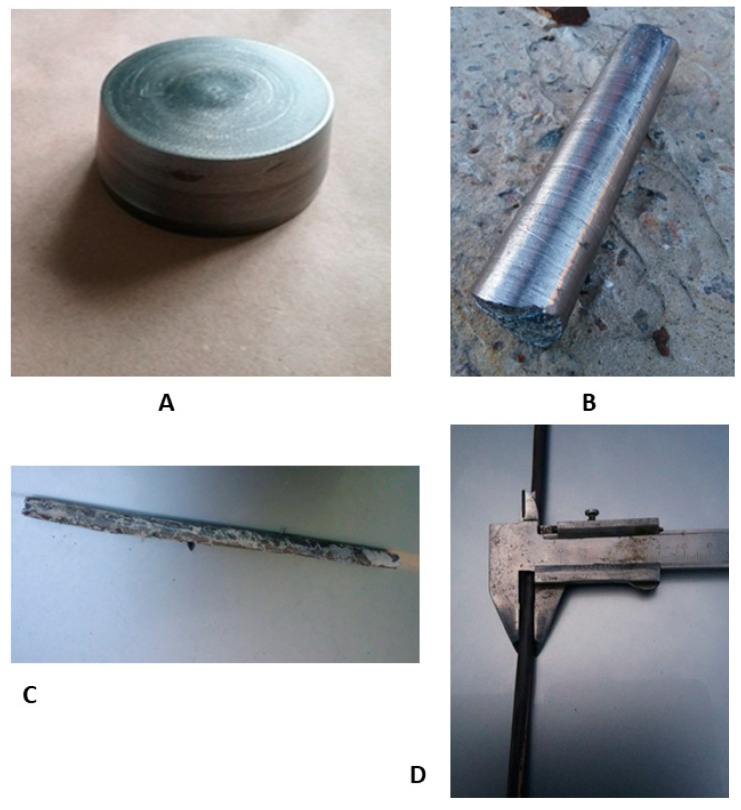
Process of Zr-Ti-Nb bar manufacturing: ingot after mechanical turning (**A**), after the forging (**B**), cross-helical rolling (**C**), and final longitudinal rolling (**D**).

For the production of the final bar for dental implant manufacturing, obtained ingots were turned onto their side surface, bottom, and top with the base diameter of 72 mm, the top diameter of 64 mm, height of 37 mm, and weight of 790 g (Figure 2A). Subsequent forging of ingots (Figure 2B) was carried out on a hammer through a square profile with heating in an oven to 800 °C. Rolling to a diameter of 18 mm (Figure 2 C) was carried out on a three-roll 10-30 cross-helical rolling mill in 6 passes heated to 800 °C. The rolling in the longitudinal rolling mill was heated to 400–600 °C, according to the scheme: diameter 18 mm → square 16 × 16 mm → oval 12.7 mm → square 12 × 12 mm → oval 8 × 6 mm → diameter 7 mm. After longitudinal rolling, heat treatment was carried out at 600 °C to align the bar (Figure 2D). Heat treatment was carried out in order to "soften" the sample to align the rod, and the temperature 600 °C was chosen to relieve stress. In this way, both processes—alignment and annealing—were provided.

As a final result of the post-cast treatment, we obtained a longitudinal bar with a length of 1.5 m and diameter of 6.2 mm. The curvature of the bar did not exceed 0.5% of its length.

### 3.2. Chemical and Mechanical Parameters

The chemical composition and mechanical properties of the new alloy (made from different master alloys—charge 1 and charge 2) and obtained bar are shown in Table 4. It should be noted that using different master alloys resulted in the same chemical composition and mechanical properties in the final Zr-Ti-Nb alloy, so CTZ-110 can be the alloy of choice for Zr-Ti-Nb alloy production, which can decrease the final price. The chemical composition fully meets the planned expectations and determines the mechanical properties. The tensile strength of the new alloy was significantly lower than a Grade 5 Ti alloy, but after post-casting treatment, it rose to 900.1 MPa, which is the same as that for standard materials used for dental implant production. The yield strength of the new materials was significantly lower than that of Ti, but it should predict reversible deformation in the final dental implant.

**Figure 3:**
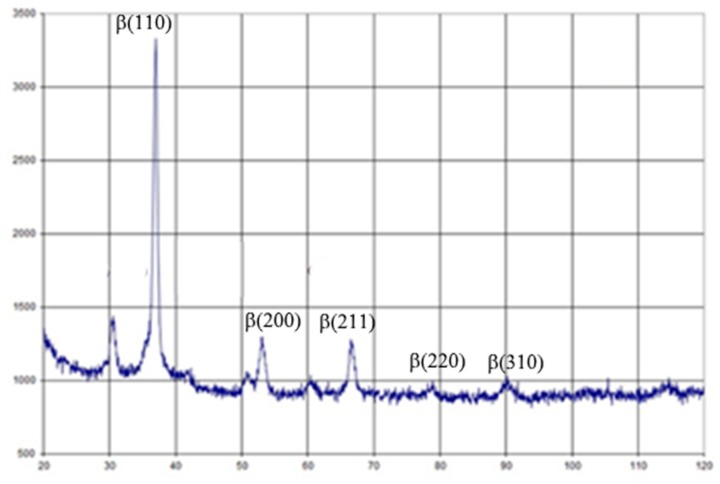
X-Ray diffraction of the Zr-Ti-Nb alloy.

The elastic modulus of the new alloy varied between 27.27 and 28.32 GPa, which is the lowest known Young modulus used for dental and orthopedic implant production [17]. Alloy post-treatment led to a slightly increasing elastic modulus up to 37.67 GPa, but is still near the mechanical parameter of natural cortical bone. Post-casting treatment led to decreasing metal elongation, but was not significantly different from that of standard Ti alloys [18]. Observing the data of X-ray diffraction shown in Figure 3 and using the Wegard rule, it was found that about 65%-66% of the alloy was a volume-centered zirconium phase. Therefore, the test alloy can be attributed to β-zircon. There are a lot of data about positive bone responses and the long-term integration of β-Ti alloys with no toxic metals (Al or V), which would allow these implants to be used in clinical cases [19]. Some research has demonstrated better late postoperative responses to β-Ti implants due to stress shielding reduction [20]. Finite element analysis of a one-piece zirconia implant was shown to be less prone to peri-implant bone overloading and subsequent bone loss in high-stress areas, especially in the labial-cervical region of the cortical bone [21]. However, additional experimental and clinical evidence and more investigations of β-zircon alloys for dental implant manufacturing are still required.

It should be noted that the existing mechanical properties of the Zr-Ti-Nb alloy in the cast state open perspectives for implant manufacturing using simpler methods, without the use of additional complex and expensive deformation processing. The existing experience of powder produced from ingots for additive technologies [22] suggests the technical feasibility of producing powders from new experimental β-zirconium alloys. Taking into account the active development of bioinspired dental and orthopedics implants with mechanical properties close to those of human bones, we can recommend new the Zr-Ti-Nb alloy as a material for additive technologies. β-alloys can provide better bone responses compared to conventional Ti-alloys after 3D scaffold implantation [23]. The initial low Young modulus of the Zr-Ti-Nb alloy, in combination with additive manufacturing techniques, can provide an ideal solution for large implant manufacturing for orthopedics.

**Figure 4:**
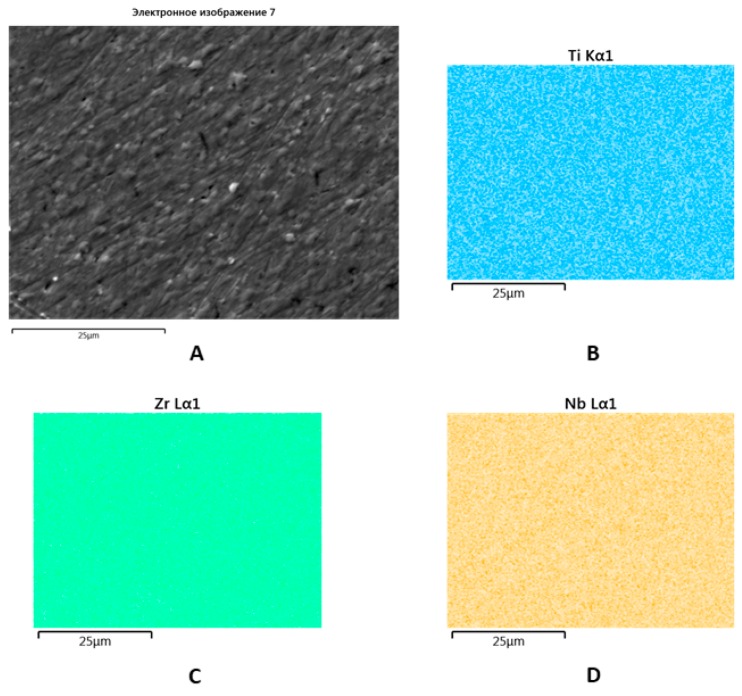
SEM image of the Zr-Ti-Nb ingot (**A**) and spectral distribution of Ti (**B**), Zr (**C**), and Nb (**D**).

SEM with EDX showed a uniform distribution of Zr, Ti, and Nb within the new alloy that made triple re-melting and further post-cast treatment possible (Figure 4).

**Figure 5:**
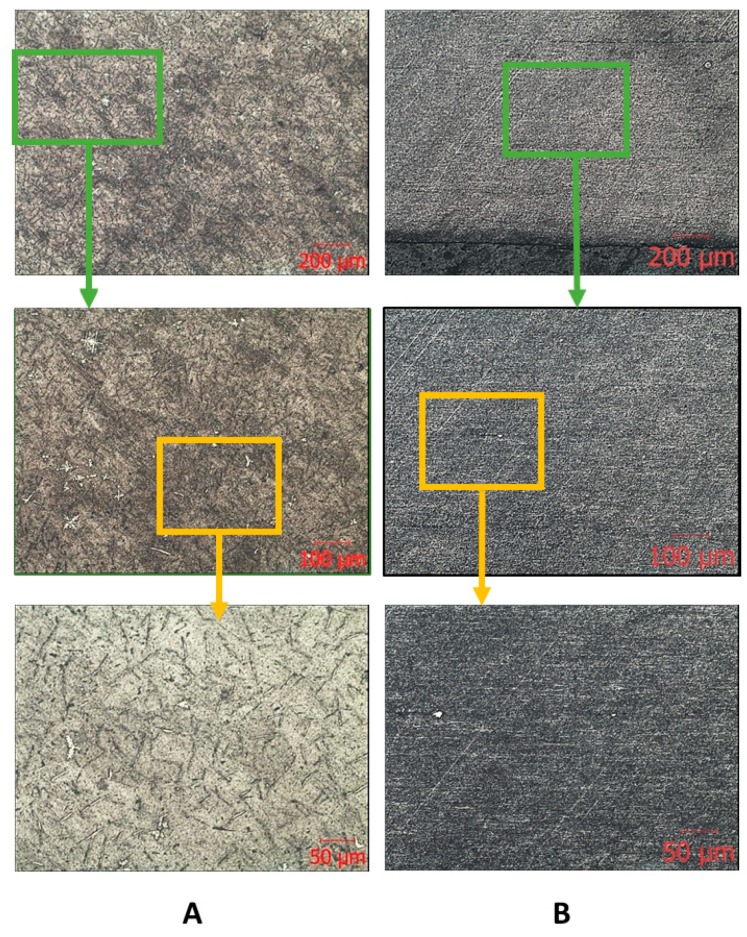
Microscopic images of the ingot (**A**) and bar (**B**) after longitudinal rolling.

**Figure 6:**
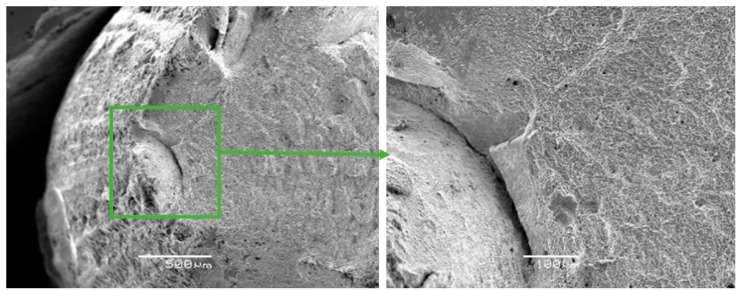
Fractographs of the fracture surface of the Zr-Ti-Nb alloy cut from the ingot.

**Figure 7:**
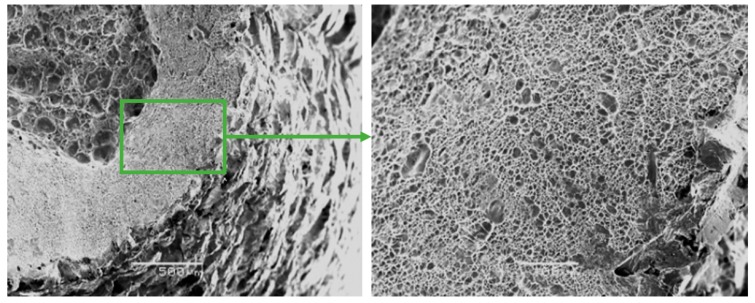
Fractographs of the fracture surface of the Zr-Ti-Nb alloy cut from the bar after final longitudinal rolling.

The results of metallographic analysis and fractograms are shown in Figure 5, Figure 6 and Figure 7. The microstructure of the Zr-Nb-Ti cast alloy is represented by β-grains that are 150–200 μm in size (Figure 5A). Forging and rolling deformation led to a change in the size of the grains, up to the complete absence of their boundaries (Figure 5B). An analysis of the samples’ microstructure (with the initial cast and in a deformed state) showed that, after deformation, the original grains were bent and stretched in the metal flow direction. The grains were parallel to this direction, and the structural changes occurred in the entire volume of the workpiece (Figure 5B).

The fracture surfaces of Zr-Ti-Nb ingot specimens after the tensile test had a cup-shaped shape that is typical for ductile fracture, with a fibrous middle (bottom) part and a smoother conical surface (bevels) (Figure 6). The bottom part had a rounded shape and occupied an area of 1.5 × 2.0 mm. The width of the conical surface formed by the mechanism of the viscous destruction characterized the ability of the material for plastic deformation to be around 0.5-1.0 mm. Kim K.M. have shown that increasing the Zr in a Ti-Zr-Nb alloy leads to the formation of a β-phase and Ti-14Nb-18Zr combination with a low modulus and sufficient plastic deformation [24]. Low-modulus alloys have shown better osseointegration in the early postoperative period and a sufficient bone quality in late regeneration terms during in-vivo experiments [25].

The fractographic analysis of fractured surfaces revealed that the fracture of Zr-Ti-Nb alloy specimens was ductile, both in the initial state (cast) and after deformation (Figure 7). Our analysis did not show areas of brittle fractures, pores, and other defects, which indicates the high quality of deformation processing. The destruction of the Zr-Ti-Nb alloy occurred by the mechanism of micropore fusion. Previous research has shown that such a mechanism can provide superior mechanical properties during implant exploitation and prevent late postoperative complications, such as the stress shielding effect [26].

### 3.3. Biocompatibility Test

**Figure 8:**
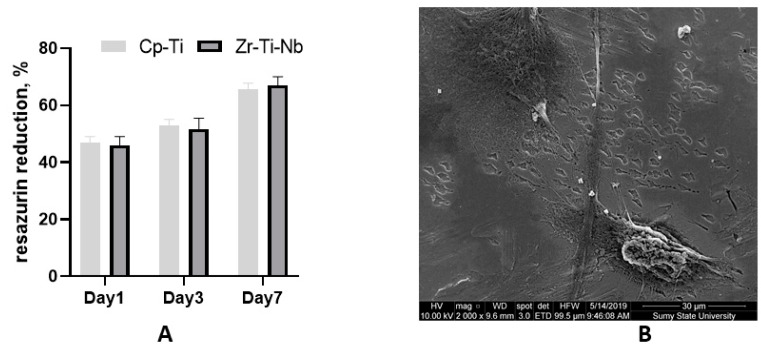
Resazurin reduction assay after osteoblast seeding on the Cp-Ti and Zr-Ti-Nb alloy (**A**) and an SEM image of osteoblasts on the Zr-Ti-Nb alloy on day 7 (**B**).

Commercially pure Ti (Cp-Ti) was used as known biocompatible material (reference). The cell culture experiment demonstrated adequate osteoblast adhesion on day one on both Cp-Ti and Zr-Ti-Nb alloys, with later proliferation on day 3 and 7. These data suggest biocompatibility and the absence of cell toxicity. SEM on day 7 demonstrated that cells adhered to the polished surface by long and short processes and cell-cell communication (Figure 8). The addition of Zr and Nb in the Ti alloy showing a high biocompatibility as a cast and after sandblasting has demonstrated a good biocompatibility in a previous study [27,28]. As mentioned above, toxic Al and V metal can prevent late material toxicity after implantation.

## 4. Conclusions

The new Zr-Ti-Nb alloy developed using the vacuum-arc melting process with further forging-rolling treatment demonstrated an extremely low Young modulus with adequate tensile and yield strength and elongation parameters. The size reduction of structural components as a result of the deformation processing of the cast material provided an increase of mechanical properties up to 26%-60%. The mechanical properties of the new alloy in the deformed state correspond to grade-5 Ti, but the elastic modulus is much closer to that of natural bone. The different master alloys used for Zr-Ti-Nb production did not affect the chemical compound and mechanical parameters, so it would be possible use affordable raw materials that decrease the final price of the new product. The cell culture experiment demonstrated full biocompatibility, indicating that the new alloy could be used for dental and orthopedics implant production.

## Figures and Tables

**Table 1:** The composition of the zirconium master alloys (main alloying elements).

Type of Zr	Mass Fraction of Impurities,%											
	N	O	C	Fe	S	Ni	Cl	Al	Ca	Mn	Ti	Cr
**Zr-I**	0.005	0.05	0.008	0.03	0.008	0.02	-	0.005	0.02	0.001	0.005	0.02
**CT** **Z** **-1** **10**	0.006	0.14	0.02	0.03	0.01	0.01	0.003	0.005	0.01	0.001	0.007	0.005

**Table 2:** The composition of the titanium master alloys.

Type of Ti	Mass Fraction of Impurities,%						
	N	O	C	Fe	Si	Ni	Cl
**TG-90**	0.02	0.04	0.02	0.05	0.01	0.04	0.08
**TG-120**	0.02	0.06	0.03	0.11	0.02	0.04	0.08

**Table 3:** The composition of the niobium master alloy.

	Mass Fraction of Impurities,%						
	N	O	C	Fe	Si	Ta	Ti
**Niobium (NbSh)**	0.02	0.06	0.03	0.11	0.02	0.04	0.07

**Table 4:** Chemical composition (EDX) and mechanical properties of the Zr-Ti-Nb alloy and resulting bar.

Samples	Chemical Composition, Mass%			Mechanical Properties				
	Ti	Zr	Nb	σ_B_, MPa	σ_O2_, MPa	E, GPa	δ, %	ψ,%
**Charge 1**	19.0	59.5	21.4	687.5	648.9	28.3	13,2	40,8
**Charge 2**	19.0	59.5	21.4	568.1	552.7	27.2	12,0	53,1
**Bar**	19.0	59.5	21.4	850.0-900.1	695,0	37.6	10.5	36,0
